# A Preliminary Study on the Potential of Manuka Honey and Platelet-Rich Plasma in Wound Healing

**DOI:** 10.1155/2012/313781

**Published:** 2012-12-04

**Authors:** Scott A. Sell, Patricia S. Wolfe, Andrew J. Spence, Isaac A. Rodriguez, Jennifer M. McCool, Rebecca L. Petrella, Koyal Garg, Jeffery J. Ericksen, Gary L. Bowlin

**Affiliations:** ^1^Physical Medicine and Rehabilitation Service, Hunter Holmes McGuire VA Medical Center, Richmond, VA 23249, USA; ^2^Department of Biomedical Engineering, Virginia Commonwealth University, Richmond, VA 23284, USA; ^3^Department of Biology, Virginia Commonwealth University, Richmond, VA 23284, USA; ^4^Department of Nursing, Old Dominion University, Norfolk, VA 23529, USA; ^5^Department of Physical Medicine and Rehabilitation, Virginia Commonwealth University, Richmond, VA 23298, USA

## Abstract

*Aim*. The purpose of this study was to determine the *in vitro* response of cells critical to the wound healing process in culture media supplemented with a lyophilized preparation rich in growth factors (PRGF) and Manuka honey. *Materials and Methods*. This study utilized cell culture media supplemented with PRGF, as well as whole Manuka honey and the medical-grade Medihoney (MH), a Manuka honey product. The response of human fibroblasts (hDF), macrophages, and endothelial cells (hPMEC) was evaluated, with respect to cell proliferation, chemotaxis, collagen matrix production, and angiogenic potential, when subjected to culture with media containing PRGF, MH, Manuka honey, and a combination of PRGF and MH. *Results*. All three cell types demonstrated increases in cellular activity in the presence of PRGF, with further increases in activity seen in the presence of PRGF+MH. hDFs proved to be the most positively responsive cells, as they experienced enhanced proliferation, collagen matrix production, and migration into an *in vitro* wound healing model with the PRGF+MH-supplemented media. *Conclusion*. This preliminary *in vitro* study is the first to evaluate the combination of PRGF and Manuka honey, two products with the potential to increase regeneration individually, as a combined product to enhance dermal regeneration.

## 1. Introduction 

The dermal healing response is a multistep process (inflammation, granulation tissue proliferation, epithelialization, and remodeling of the wound site) which may result in a number of different outcomes: complete healing, scarred healing, or a chronic nonhealing wound [1, 2]. 

In nonhealing wounds, such as pressure and diabetic ulcers, the carefully coordinated wound healing process has been altered. Neutrophils accumulate in the wound site and leave the wound stuck in a state of chronic inflammation. While inflammation normally resolves within 1-2 days as neutrophil number decreases, the prolonged presence of these cells contributes to a disordered network of regulatory cytokines. This aberrant set of regulatory signals has far-reaching effects on all the cells involved in dermal healing (macrophages, fibroblasts, etc.) and results in increased proteolytic activity and improper extracellular matrix (ECM) deposition. In order for these wounds to heal, the self-propagating loop of chronic inflammation must be disrupted. Current clinical treatments often center around surgical debridement, exudate management, and minimization of bacterial adherence (biofilm) to remove inflammatory stimuli. Other treatments involve the utilization of recombinant growth factors, synthetic protease inhibitors, or pH modifying ointments [1, 2]. However, to date, there has been no single treatment that has proven to be optimal at stimulating the resolution of chronic wounds, and the future may lie in regenerative medicine's ability to modify cellular behavior within the wound site.

Honey had been used medicinally for centuries, due to its inherent wound healing capacity. However, the introduction of penicillin significantly reduced its role [3–8]. Recently, with the emergence of antibiotic-resistant bacteria and a better scientific understanding of how honey influences healing, honey (specifically active *Leptospermum* honey from New Zealand, known as Manuka) has once again become an acceptable product in the treatment of wounds. The major benefit of Manuka honey lies in its potent antibacterial properties. Honey has a high osmolarity and a high sugar content, the combination of which has been shown to inhibit microbial growth [5, 9, 10]. Manuka honey is also known to have a relatively low pH (3.5–4.5), which, in addition to inhibiting microbial growth, will stimulate the bactericidal actions of macrophages, and in chronic wounds reduce protease activity, increase fibroblast activity, and increase oxygenation [5, 10–12]. Hydrogen peroxide is slowly released from honey placed on a wound through the interaction of wound exudates with the honey's inherent glucose oxidase. This hydrogen peroxide is in sufficient concentration to be antibacterial, yet dilute enough to be nontoxic while promoting fibroblast proliferation and angiogenesis [3, 5, 9, 10, 12]. Manuka honey also possesses nonperoxide antibacterial activity in what is called the Unique Manuka Factor (UMF) due to the presence of methylglyoxal [6, 12].

Honey has been shown to contain a number of phenolic compounds, which are known to scavenge and remove reactive oxygen species (ROS) released by neutrophils [6]. Leong et al. [6] demonstrated that honey can suppress oedema and leukocyte infiltration in a mouse model of neutrophilic inflammation. Tonks et al. [7] demonstrated that monocytes cultured in the presence of honey were stimulated to produce a number of pro and anti-inflammatory cytokines (tumor necrosis factor alpha (TNF-*α*), interleukin-1 beta (IL-1*β*), and interleukin-6 (IL-6)) and may indicate modulation towards resolution in nonhealing wounds.

Platelet-rich plasma (PRP) therapy has been gaining momentum as a bedside regenerative medicine procedure and has been used to stimulate regeneration of osteochondral defects [13–15], tendon/ligament injuries [13–19], and chronic dermal wounds (diabetic and pressure ulcers) [14, 15, 20, 21] in clinical studies. PRP is a simple and cost-effective method for collecting and concentrating autologous platelets (some clinical studies have published on the use of pooled-banked PRP in order to overcome donor variability with no evidence of immunoreactions [22–25]) for the purpose of activating and releasing their growth factor-rich alpha and dense granules. These granules releases a number of growth factors and cytokines, including: platelet-derived growth factor (PDGF), transforming growth factor beta (TGF-*β*), vascular endothelial growth factor (VEGF), fibroblast growth factor (FGF), epidermal growth factor (EGF), and others [13, 15, 16, 20, 26–28]. These listed factors, in conjunction with the numerous factors contained in PRP not listed, are known to accelerate cell migration and proliferation, promote ECM production, as well as play a role in macrophage phenotype and inflammation resolution [13, 26, 27, 29–33].

There is currently no consensus as to how PRP should be most effectively utilized in the treatment of wounds. There have been a number of methods reported on delivering PRP; most involve the creation of an activated-platelet gel with thrombin [13–15, 34] or CaCl_2_ [13–15, 28]. These PRP gels are easily applied to wound sites through injection or topical application. However, studies have shown that the use of these PRP gels are inefficient due to the rapid release and diffusion of the factors [13]. Several techniques have been evaluated for sustained release, including gelatin gel microspheres [35], lyophilized PRP [36–39], and alginate beads [40]. Collectively, these studies demonstrated the importance of keeping preparations rich in growth factors (PRGF) in the wound site and slowly activating/releasing them as the wound site becomes infiltrated with reparative cells.

The purpose of this study was to determine the *in vitro* response of three cell types critical to wound healing (fibroblasts, endothelial cells, and macrophages) when subjected to culture media supplemented with Manuka honey, a powdered PRGF (a lyophilized version of PRP), or a combination of Manuka honey and PRGF. The hypothesis being that Manuka honey and PRGF will increase cellular activity over control media, with a corollary that the combination of honey and PRGF will provide the greatest increase due to increased growth factor and cytokine activity through acid activation. Manuka honey has been documented to have an acidic pH, and factors such as TGF-*β* are known to become physiologically active when subjected to an acid treatment.

## 2. Materials and Methods

### 2.1. Creation of PRP/PRGF and Honey Media

PRP and PRGF were created using previously described methods [41, 42]. Briefly, fresh human whole blood from 3 donors was purchased (Biological Specialty Corp., Colmar, PA, USA), pooled, and used in a SmartPReP 2 (Harvest Technologies Corp., Plymouth, MA, USA) centrifugation system to create PRP per manufacturers protocol. PRP was then subjected to a freeze-thaw-freeze (FTF) cycle in a −70°C freezer for cell lysis (centrifuge tubes containing PRP were placed in a −70°C freezer for 24 hrs followed by a 37°C water bath for 1 hr, and then returned to the −70°C freezer for 24 hrs). Frozen PRP was then lyophilized for 24 hrs to create a dry PRGF powder which was finely ground in a mortar and pestle prior to use. This dry PRGF powder was added to the test media used in this study as a weight percentage (w/w %).

 The honeys used in this study were a pure Manuka honey (Wedderspoon Organic, UMF 16+, Vancouver Island, BC, Canada) and Medihoney (MH, DermaSciences Inc., Princeton, NJ, USA), which is a medical grade, sterile, Manuka honey that has been filtered to remove any potential residual pollen or bee byproducts. Honeys were added to test medias as a volume percentage (v/v %).

### 2.2. Western Blot Analysis of Honey-Activated PRP

It has been previously documented that TGF-*β* can be transformed from a nonactive state to a physiologically active form through pH modification (TGF-*β* ELISA instructions). To determine what, if any, activation potential the honey (pH known to be 3.5–4.5) afforded, PRP was mixed in varying ratios (1 : 1, 1 : 5, 1 : 10, 5 : 1, and 10 : 1) with MH and subjected to a modified Western Blot to determine physiologically active TGF-*β* through fluorescence. Briefly, the PRP : MH solutions were blotted on a PVDF membrane, along with PRP that was acid activated using a modified TGF-*β* ELISA protocol (Promega, Madison, WI, USA). The membrane was blocked in Odyssey Blocking Buffer for one hour at room temperature. After blocking, samples were incubated in anti-human TGF-*β* antibody (Promega, Madison, WI, USA) at room temperature for 1.5 hours. All samples were then washed four times with 0.1% Tween-20 in PBS, after which the signal from anti-human TGF-*β* antibody was detected with goat anti-mouse IgG secondary antibody tagged with a fluorescent 800 nm marker (Thermo Scientific, Waltham, MA, USA). To account for antibody background fluorescence, each sample was also incubated with secondary antibody only. Samples were incubated in the secondary antibody for 1 hour at room temperature without exposure to light. After washing, the samples were scanned using the 800 nm channel of the Odyssey Infrared Imaging System (LI-COR Biosciences, Lincoln, NE, USA) at an intensity of 3.5. Fluorescence intensities were measured using circular gates that completely surrounded each sample. Background fluorescence that was obtained from samples incubated with secondary antibody only was subtracted from the signal intensities of the samples incubated with both primary and secondary antibodies.

### 2.3. Effect of PRGF and Honey on Cell Proliferation

Human dermal fibroblasts (hDF) were seeded subconfluently in a 48-well plate at 50,000 cells/well in 500 *μ*L of control media (DMEM/F12 supplemented with 10% fetal bovine serum (FBS), 1% penicillin/streptomycin (P/S)). Following adhesion of cells to the well-plate (~2 hrs) control media was replaced with test media (control media supplemented with Manuka honey in 0.1, 1, 5, 10, or 20% v/v, MH in 0.1, 1, 5, 10, or 20% v/v, PRGF in 0.1, 1, 5, or 10 mg/mL or a combination of 0.1% MH and 1 mg/mL PRGF). Test media was changed on days 1, and 4, with an MTS assay (CellTiter 96 Aqueous Non-Radioactive Cell Proliferation Assay, Promega, Madison, WI, USA) performed on days 1, 4, and 7 to determine mean cell count.

Using a protocol nearly identical to that of the hDF proliferation study, immortalized human pulmonary microvascular endothelial cells (hPMEC, donated generously from Dr. C. J. Kirkpatrick) were seeded subconfluently at 25,000 cells/well in a 48-well plate in 500 *μ*L of control media (M199 media supplemented with 10% FBS, 1% P/S, Glutamax 100x, G418, heparin sodium salt (50 *μ*g/mL), and endothelial cell growth supplement (ECGS, 50 *μ*g/mL)). Following adhesion of cells to the well-plate (~2 hrs) control media was replaced with test media (control media supplemented with Manuka honey in 0.1, 1, 5, 10, or 20% v/v, MH in 0.1, 1, 5, 10, or 20% v/v, PRGF in 0.1, 1, 5, or 10 mg/mL or a combination of 0.1% MH and 1 mg/mL PRGF). Test media was changed on days 1, and 4, with an MTS assay performed on days 1, 4, and 7 to determine mean cell count.

Human peripheral blood macrophages (ATCC, CRL9855) were seeded subconfluently in a 48-well plate at 50,000 cells/well in 500 *μ*L of control media (RPMI 1640, supplemented with 10% FBS, 1% P/S). Following adhesion of cells to the well-plate (~2 hrs) control media was replaced with test media (control media supplemented with either Manuka honey or MH in 0.1, 1, 5, 10, or 20% v/v). Test media were changed on days 1, 4, and 7, and an MTS assay was performed on the same time points to determine a mean cell count. Changed media was retained and used for subsequent ELISA analysis to determine what role the honey may play in stimulating macrophage inflammation. PRGF supplemented media was not investigated with this cell type as previous work has demonstrated that the presence of PRGF had little impact on macrophage proliferation [42].

### 2.4. Macrophage Inflammation Response

Using the retained media from the proliferation study, ELISAs were run per manufacturer's protocol to determine the inflammatory response of macrophages to either pure Manuka honey or MH. ELISAs were conducted to determine levels of TNF-*α* (Antigenix America Inc., Huntington Station, NY, USA), an indicator of inflammation and M1 phenotype, Interleukin-10 (IL-10, Peprotech, Rocky Hill, NJ, USA), an interleukin commonly associated with inflammation resolution and a regulatory phenotype, and VEGF (Antigenix America Inc.), which is critical to angiogenesis and is expressed by M2 macrophages.

### 2.5. Effect of PRGF and Honey on Cell Chemotaxis

Similar to the aforementioned cell proliferation studies, cell chemotaxis studies were conducted on both macrophages and hPMECs (hDF chemotaxis was evaluated with an *in vitro* wound healing assay) using a transwell plate and an MTS assay. Human macrophages were seeded in the top insert of a transwell plate (8 *μ*m diameter pores, Corning Inc., Lowell, MA, USA) with 50,000 cells/well in 200 *μ*L control media, while the bottom insert was filled with 600 *μ*L of test media (control media supplemented with Manuka honey in 0.1, 1, 5, 10, or 20% v/v, MH in 0.1, 1, 5, 10, or 20% v/v, PRGF in 0.1, 1, 5, or 10 mg/mL or a combination of 0.1% MH and 1 mg/mL PRGF). Media from both the top insert and the bottom well were changed on days 1, and 2, and the cells were counted using an MTS assay on day 3. The MTS assay was performed on both inserts and the bottom wells to distinguish between cell chemotaxis and proliferation. This study was then repeated with hPMECs.

### 2.6. *In Vitro* Wound Healing Assay

An *in vitro* wound healing assay was performed to determine the rates of migration of hDFs into a wound site, simulating hDF migration into dermal wounds *in vivo* [43]. A 48-well plate was coated with fibronectin (10 *μ*g/mL) and blocked with bovine serum albumin (2 mg/mL) prior to cell seeding. The exterior underside of each well was marked with a permanent marker to serve as a reference line. Each well was then seeded to confluency with 100,000 hDFs in a standard DMEM/F12 control media (containing 1% P/S, and 2% FBS to minimize cellular proliferation). The cells were allowed to attach and spread overnight, creating a completely uninterrupted layer of cells. After 18 hrs, a 200 *μ*L pipette tip was scraped across the well, perpendicular to the reference line to create an intersection point, to create the *in vitro* wound. The wells were gently washed with control media to remove any dislodged cells, and test media was added. Test media consisted of control media supplemented with 1% v/v MH, 1 mg/mL PRP, and a combination of 1% v/v MH and 1 mg/mL PRP (chosen based upon results of hDF proliferation study). Images were taken at time 0, 6, 12, 18, and 30 hr using an inverted Nikon microscope with a 4x objective and color camera. Using the reference intersection point, images were taken at the same location at each successive time point. Using image analysis software (ImageTool), the area of each wound was measured, and a rate of healing was determined.

### 2.7. Hydroxyproline Assay

To determine the amount of ECM produced by the hDFs, a hydroxyproline assay was performed based upon previously published protocols [44–46]. Briefly, hDFs were seeded near confluency at 100,000 cells/well in a 48-well plate in 500 *μ*L of control media. Following adhesion of cells to the well-plate (~2 hrs) control media were replaced with test media (control media supplemented with 1% MH v/v, 1 mg/mL PRGF, or a combination of 1% MH and 1 mg/mL PRGF). These specific test media were chosen for evaluation based upon results of previous hDF experiments conducted in this study. Media was changed every other day and retained for assessment with the assay on days 1, 7, 14, 21, and 28. Retained media was hydrolyzed in 100 *μ*L of 6 M hydrochloric acid in a boiling water bath and subsequently lyophilized until dry. Dry samples were diluted in 50 *μ*L of distilled water, and 450 *μ*L of chloramine T reagent were added and allowed to oxidize in the dark at room temperature for 25 minutes. 500 *μ*L Ehrlich's reagent added to each sample, mixed gently, and incubated for 20 minutes at 60°C. 100 *μ*L of each sample were then transferred to a 96-well plate and read at 570 nm on a spectrophotometer (SpectraMax Plus, Molecular Devices, Sunnyvale, CA, USA).

### 2.8. *In Vitro* Bead Angiogenesis Assay

A preliminary *in vitro* angiogenesis bead assay was performed, similar to that of Chen, et al. [47]. After Cytodex microcarrier beads (Sigma-Aldrich, St. Louis, MO, USA) were autoclaved and hydrated overnight, 10 *μ*L of bead solution was placed in a 15 mL tube and rinsed 3 times with complete hPMEC medium before being mixed with approximately 1 million hPMEC. The tube was incubated in standard conditions (37°C, 5% CO_2_), with inversion of the tube occurring every half hour over 3 hours to ensure cells attached to the beads. The media was then removed from the centrifuge tube, placed in a flask, and incubated overnight. After incubation, beads were rinsed in PBS and transferred back to a 15 mL tube. Once the beads had settled to the bottom, any remaining media were removed, and beads were washed once more with PBS. After allowing the beads to settle to the bottom of the tube a second time, the beads with adherent cells were resuspended in a collagen gel solution (80% liquid Purecol with 10% 0.1 M NaOH and 10% 10X PBS) containing 1000 KIU/mL aprotinin (Sigma-Aldrich, St. Louis, MO, USA). Simultaneously, 250 *μ*L of collagen gel solution with aprotinin was placed in the bottom of a 24 well plate and incubated at standard conditions until gelation occurred. Once the first layer was gelled, 250 *μ*L of cell suspension was placed on the first collagen gel layer and was incubated for 2 hours to again allow for gelation. Supplemented media was then created as described previously (Manuka honey in 0.1, 1, 5, 10, or 20% v/v, MH in 0.1, 1, 5, 10, or 20% v/v, PRGF in 0.1, 1, 5, or 10 mg/mL or a combination of 0.1% MH and 1 mg/mL PRGF) and 500 *μ*L was placed on top of the gel solution containing the cell covered beads and incubated under standard conditions for 6 days. At days 1, 4, and 6, images were captured using a Nikon Eclipse TE200 microscope with a Dage-MTS digital camera. Three images were taken for each test media at each time point. To quantify the percentage of hPMEC sprouts per bead, a circular grid was overlayed onto each bead, dividing it into 36 sections. All sprouts extending from the surface of the beads were counted, summed, and divided by 36 to determine the percentage of sprouts for each bead. In addition, the length of each sprout extending from the surface of the beads was quantified using ImageTool 3.0 software (Shareware provided by UTHSCSA). Results are presented as average sprout length per bead (*n* = 3).

### 2.9. Statistical Analysis

All statistical analysis was based on a Kruskal-Wallis one-way ANOVA on ranks and a Tukey-Kramer pairwise multiple comparison procedure (*α* = 0.05) performed with the JMP IN 8.0 statistical software package (SAS Institute, Inc.). Graphical depictions of mean data were constructed with Microsoft Excel 2007, with error bars representing standard deviations from the mean. All studies were done in triplicate (*n* = 3).

## 3. Results

### 3.1. Western Blot Analysis of Honey-Activated PRP

**Figure 1:**
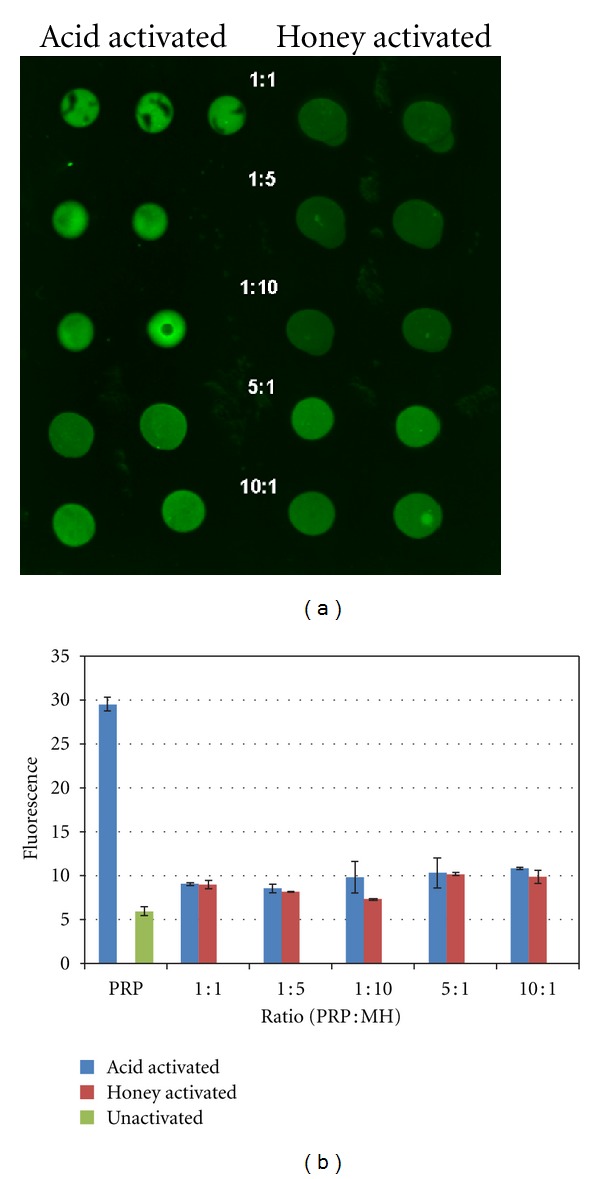
(a) Fluorescence results from TGF-*β* western blot for acid- and honey-activated PRP. All ratios are PRP : MH by volume. (b) Quantification of fluorescence from TGF-*β* Western blot.

The Western blot analysis of honey-induced PRP activation is shown in Figure 1. While the modified Western blot analysis used in this study does not provide an exact quantification of physiologically active TGF-*β*, the differences in sample fluorescence can be used to provide a general indication about the presence of TGF-*β* activated through either acid or honey treatment. Statistical analysis revealed there to be no significant differences in activated TGF-*β* regardless of whether PRP was activated through acid treatment or mixing with MH. Additionally, both the acid and MH-treated PRP was significantly more fluorescent than the unactivated PRP.

### 3.2. Effect of PRGF and Honey on Cell Proliferation

**Figure 2:**
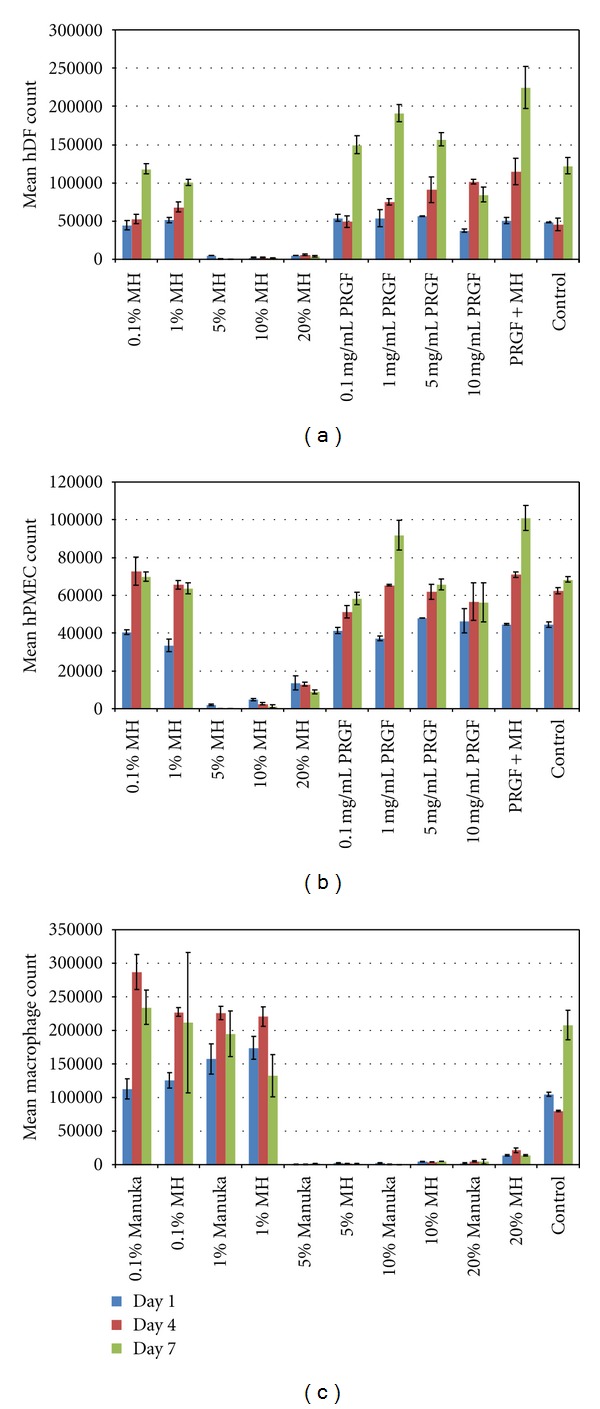
Results of mean hDF (a), hPMEC (b), and macrophage (c) proliferation from MTS assays at days 1, 4, and 7. Manuka honey results are not shown for hDF and hPMEC proliferation as they were not statistically different from MH.

Results of the hDF, hPMEC, and macrophage proliferation studies are shown in Figure 2. hDF proliferation was observed over 7 days in response to test medias containing varying concentrations of Manuka honey, MH, PRGF, and a combination of PRGF+MH, and cell numbers ranged from near 0 for the higher Manuka/MH concentrations to greater than 224,000 for the PRGF+MH on day 7. Statistical analysis revealed the combination of PRGF+MH to significantly enhance cell proliferation over all other groups at days 4 and 7. Additionally, the presence of PRGF alone had a greater impact on cell proliferation than MH alone, as 0.1, 1, and 5 mg/mL PRGF had significantly higher cell numbers than the 0.1 or 1% MH. There were no significant differences between Manuka honey and MH-supplemented media of the same concentration at any time point.

 The results of the hPMEC proliferation study were similar to those of the hDF study, with cell numbers ranging from near 0 for the higher Manuka/MH concentrations to over 101,000 for the PRGF+MH test media. However, the differences between the PRGF+MH and the other test media were not as pronounced as with the hDF study. While the PRGF+MH had the highest average cell number at day 7, it was not statistically different from the 1 mg/mL PRGF test media at day 7 (*P* = 0.1343). Additionally, there were few differences seen between test medias on day 4 as well; with the PRGF+MH, 1 and 5 mg/mL PRGF, 0.1 and 1% MH, and control all being statistically the same. As before, there were no significant differences between the Manuka honey and MH supplemented medias.

 The macrophage proliferation study was conducted without the addition of PRGF, as a previous study revealed that macrophage proliferation was not significantly influenced by the presence of PRGF [42]. Therefore, macrophage proliferation was compared between test medias containing pure Manuka honey and MH. Macrophage cell numbers ranged from near 0 for higher honey concentrations to over 286,000 for the 0.1% Manuka honey at day 4. Statistical analysis revealed there to be no significant differences between test medias of Manuka honey and MH at the same concentration. Macrophage proliferation peaked at day 4 for the 0.1 and 1% Manuka and MH test medias, at which point cells became contact inhibited and cell number decreased for day 7.

### 3.3. Macrophage Inflammation Response

**Figure 3:**
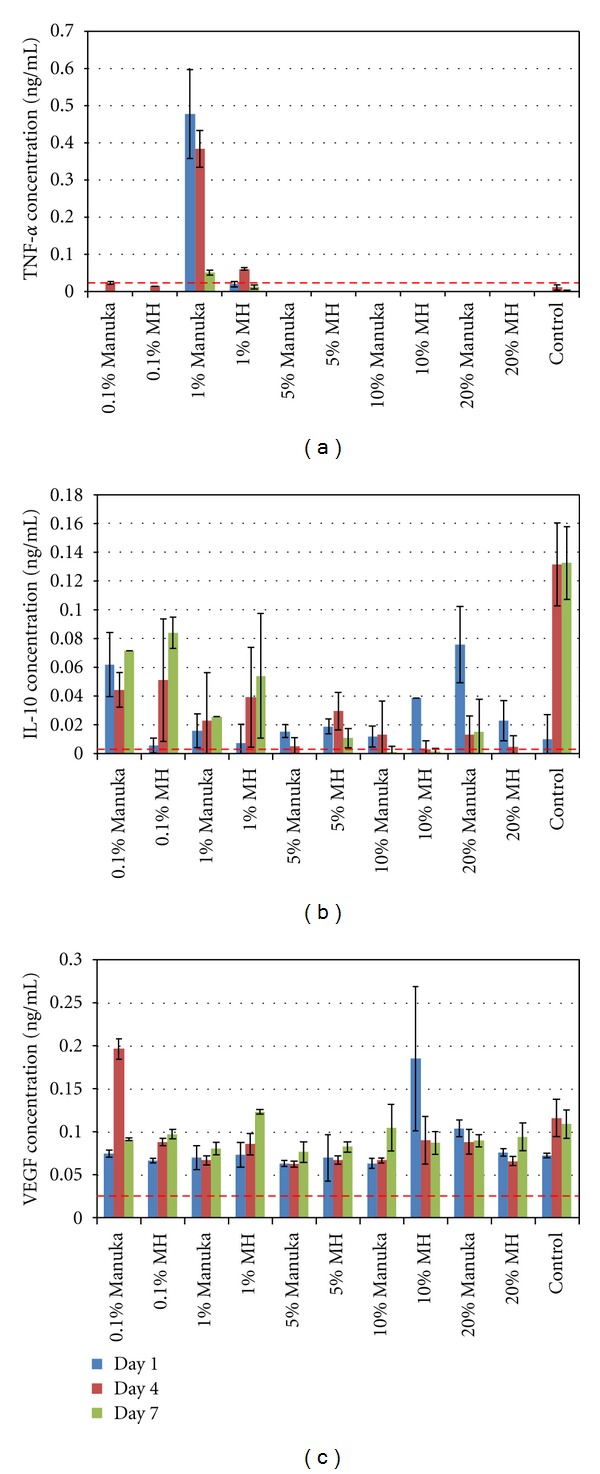
Results of the macrophage inflammation response studies, with mean TNF-*α* (a), IL-10 (b), and VEGF (c) from days 1, 4, and 7. Dashed lines indicate the minimum levels of detection of 0.031, 0.005, and 0.023 ng/mL for the TNF-*α* IL-10, and VEGF ELISAs, respectively.

Results of the macrophage inflammation response ELISAs are shown in Figure 3. Media used in the macrophage proliferation study was retained and analyzed through ELISA for the presence of TNF-*α*, IL-10, and VEGF; three cytokines that can provide insight into macrophage function and phenotype. For the TNF-*α* ELISA, the 1% Manuka test media yielded the only significant result, with a peak TNF-*α* concentration of 0.48 ng/mL on day 1 and significant decreases in TNF-*α* at days 4 and 7. The IL-10 ELISA results ranged from 0 for 5% Manuka on day 7 to 0.13 ng/mL for the control at day 7. Statistically, the control media on days 4 and 7 were significantly different from all other groups, with the exception of the 0.1% Manuka on day 7, 0.1% MH on day 7, and the 20% Manuka on day 1. The 20% Manuka day 1 results are most likely outliers as the 20% Manuka proved to be cytotoxic. While there appears to be a trend of increasing IL-10 release from the lower honey concentrations (0.1 and 1%), these increases were not statistically significant. Values of VEGF release ranged from 0.06 to 0.19 ng/mL for 5% Manuka on day 1, and 0.1% Manuka on day 4, respectively. Test media were not statistically different from one another, with the exception of the 0.1% Manuka on day 4 and the 10% MH on day 1, and these were most likely outliers as there is no logical explanation for such a random spike in VEGF production. While there does appear to be a trend of increasing VEGF over time for all samples, the differences were not significant. 

### 3.4. Effect of PRGF and Honey on Cell Chemotaxis

**Figure 4:**
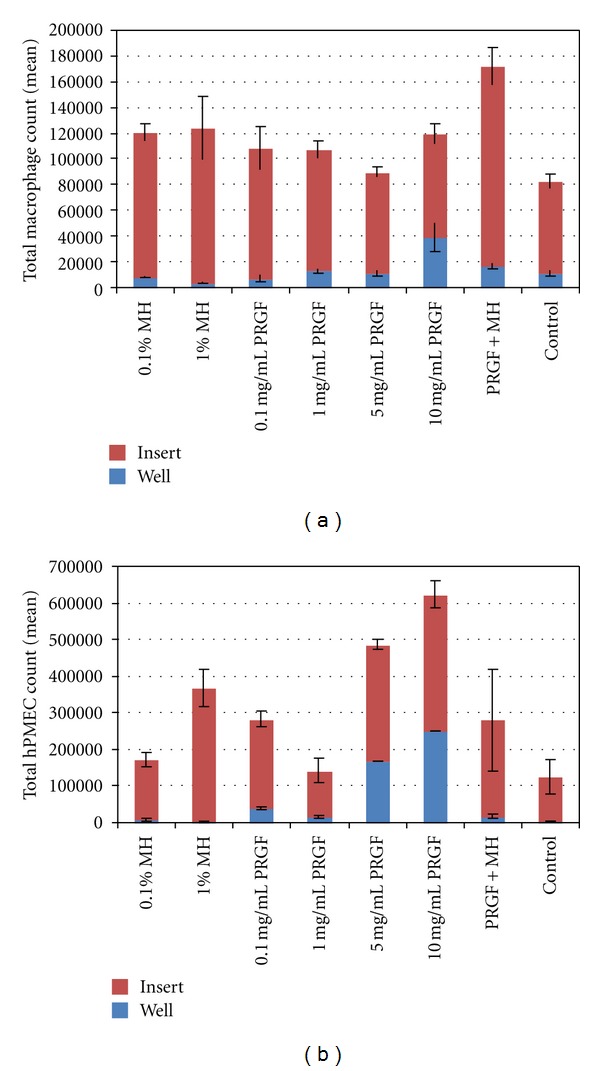
Results of macrophage (a) and hPMEC (b) chemotaxis from MTS assay quantification in both the top insert and bottom well. The results of cell chemotaxis from the addition of Manuka honey are not included as they were not significantly different from those of MH. Higher honey concentrations (5, 10, and 20% v/v) were excluded as they resulted in cell death.

The results of the macrophage and hPMEC chemotaxis are shown in Figure 4. The presence of Manuka/MH did not induce significant macrophage chemotaxis, but did increase cell proliferation in the insert at the lower MH concentrations. Similar to the results of the cell proliferation studies, there were no significant differences between the pure Manuka honey and the MH medias. The higher honey concentrations (5, 10, and 20% v/v) resulted in cell death and are therefore not included in Figure 4. The 10 mg/mL PRGF resulted in a statistically significant increase in chemotaxis to the bottom well over the control media, but did not induce a statistically significant increase in cell proliferation in the insert. The combination of PRGF+MH contributed mainly to the proliferation of macrophages, with the PRGF+MH insert experiencing the largest increase in cell number (significant over all other groups) with chemotaxis that was not significantly different from those of the control media. The 10 mg/mL PRGF formed a clot when added to the media, making it difficult to ensure accurate cell counting with the MTS. While reasonable numbers were achieved, their accuracy cannot be assured due to the clot formation and potential loss of cells.

hPMEC chemotaxis was also minimal, with only the 5 and 10 mg/mL PRGF inducing a statistically significant amount of cells to travel from the insert to the bottom well. These test media actually formed a semisolid clot, making it difficult to accurately assess cell number in the bottom well as cells seemed to readily migrate into the clot. The 1% MH, PRGF+MH, and 0.1 mg/mL PRGF all stimulated significant cellular proliferation over control media in the insert, but did not induce chemotaxis.

Future studies will utilize a longer time course for chemotaxis studies, as the 72 hrs in this study may not have been long enough to see cellular chemotaxis on a quantifiable scale.

### 3.5. *In Vitro* Wound Healing Assay

**Figure 5:**
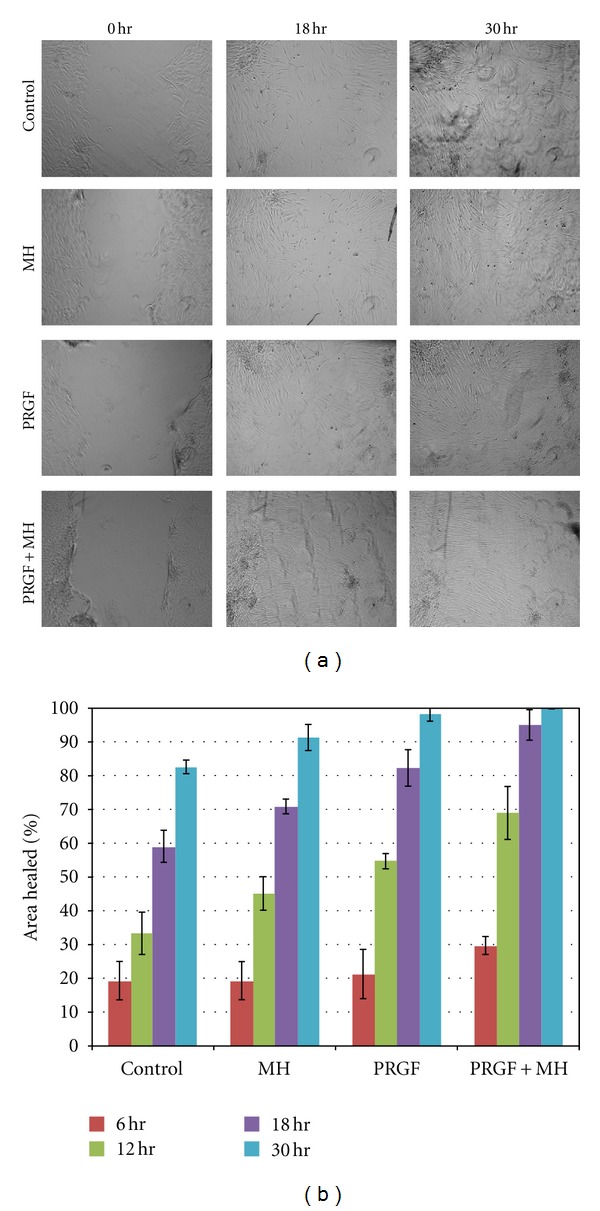
Light microscopy images of the *in vitro* wound healing assay taken at time 0, 18, and 30 hr after wounding (a). Graph of the mean % area healed for each of the test media at 6, 12, 18, and 30 hr after wounding (b).

Results of the *in vitro* wound healing assay are shown in Figure 5. The combination of PRGF+MH demonstrated more rapid hDF infiltration into the denuded area compared to all other groups at time points 12 and 18 hrs. By the 30 hr time point, the wounded area in the PRGF+MH group was indistinguishable from surrounding areas, whereas the other groups still contained small areas of exposed culture plastic. However, while there was a trend towards increased healing with the PRGF+MH over all other medias, these differences were not all statistically significant due to a limited sample population. The PRGF+MH exhibited significantly more healing than the controls at 30 hr, but was not significantly different from the PRGF or the MH medias at the same time point. At the 18 hr time point both the PRGF+MH and PRGF were significantly different from control media, while not statistically different from one another. The largest jump in area healed occurred with the PRGF+MH at the 12 hr time point (69% area healed compared to 33% for control), which was significantly different from all other medias at that time.

### 3.6. Hydroxyproline Assay

**Figure 6:**
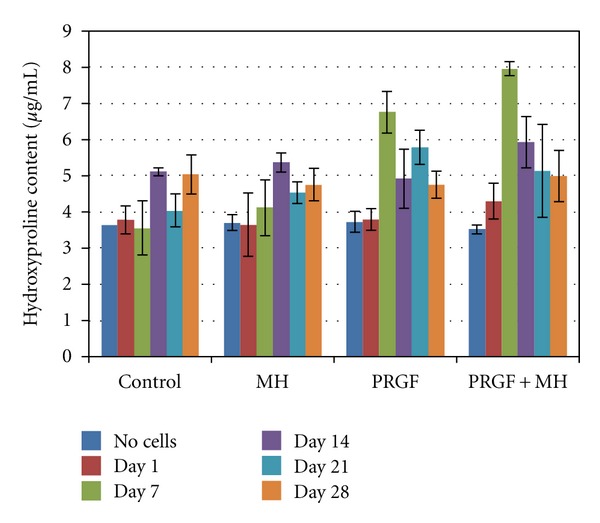
Results of the hydroxyproline assay performed on retained media on days 1, 7, 14, 21, and 28.

The results of the hydroxyproline assay are shown in Figure 6. Mean hydroxyproline concentrations ranged from 3.5 *μ*g/mL for the PRGF+MH with no cells to 7.9 *μ*g/mL for the PRGF+MH on day 7. Results indicate that hydroxyproline, and therefore collagen, content peaked on day 7 for the PRGF and PRGF+MH and then decreased to an almost steady concentration, potentially due to cell proliferation resulting in contact inhibition and apoptosis or loss of aggregated collagen through subsequent media changes. Statistical analysis revealed the PRGF and PRGF+MH to be statistically the same at the day 7 time point, while superior to the MH and control medias at the same time point. There were no significant differences between test medias at the other time points.

### 3.7. *In Vitro* Bead Angiogenesis Assay

**Figure 7:**
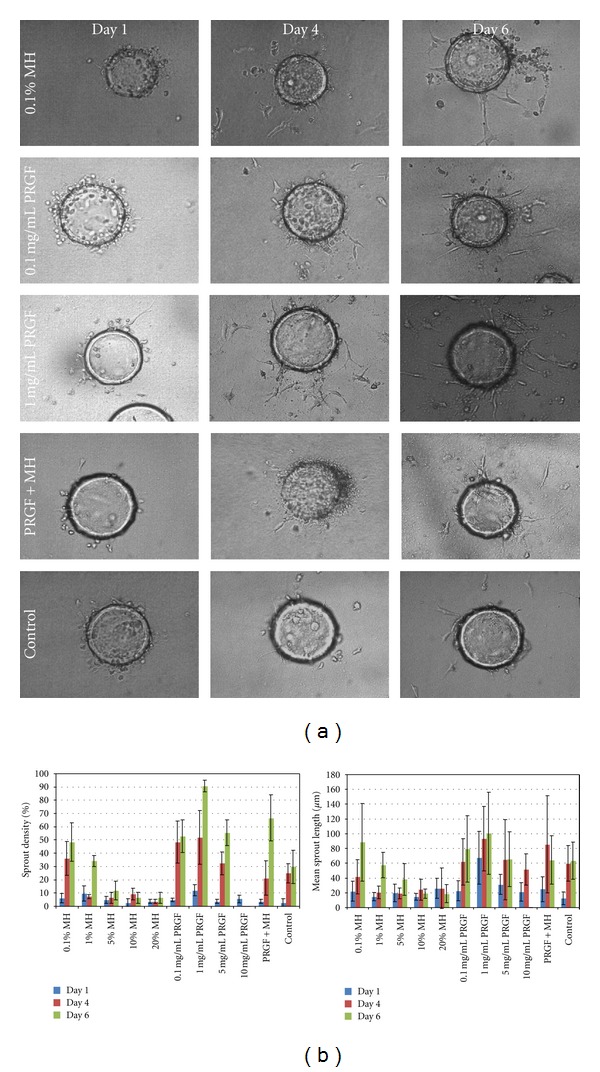
Representative light microscopy images of the *in vitro* bead angiogenesis assay performed with hPMECs cultured on Cytodex beads. The 0.1% MH, 0.1 mg/mL PRGF, 1 mg/mL PRGF, PRGF+MH, and control are shown on days 1, 4, and 6 as those test medias resulted in maximum sprout formation (a). Mean sprout densities (b, left) and mean sprout lengths (b, right) are shown for MH, PRGF, and control medias. The results of Manuka honey supplemented media are not included as they were not significantly different from those of MH. Higher honey concentrations (5, 10, and 20% v/v) were excluded from the sprout density and sprout length graphs as they resulted in cell death.

Results of the *in vitro* bead angiogenesis assay are shown in Figure 7. Mean sprout densities ranged from 2.7% for controls on day 1 to 90.7% for the 1 mg/mL PRGF on day 6. Statistical analysis revealed the 0.1 mg/mL PRGF, 1 mg/mL PRGF, and PRGF+MH to be significantly different from the control at day 6, with the 1 mg/mL PRGF and PRGF+MH not being different from each other. At day 4, only the 1 mg/mL PRGF was significantly different from the control, while none of the test medias were significantly different from the control at day 1. Mean sprout lengths ranged from 12.6 *μ*m for control on day 1 to 100.5 *μ*m for 1 mg/mL PRGF on day 6. Statistical analysis of the mean sprout length data was fairly inconclusive due to the large deviations in both sprout number and length from bead to bead, however; it was determined that the 1 mg/mL PRGF was significantly different from the control on day 6. Trends in sprout length mean tend to indicate that both sprout length and density increase with PRGF concentration. Throughout this study, the 5 and 10 mg/mL PRGF test medias suffered from gel formation which made their analysis extremely difficult and reported results unreliable. Surprisingly, the Manuka and MH supplemented medias proved to be rather unsuccessful at promoting sprout formation, as the higher concentrations (5, 10, and 20% v/v) resulted in hPMEC death and the lower concentrations failed to demonstrate increases in sprout density or length over control media.

## 4. Discussion

PRP, and the powdered PRGF used in this study, is an attempt to harness the healing potential of the platelets and their inherent growth factors to initiate and accelerate the body's normal healing response. While PRP and PRGF have been investigated previously, this study was the first attempt at enhancing their bioactivity through the addition of Manuka honey, which in and of itself has been shown to be effective in treating chronic dermal wounds in a number of clinical settings. This preliminary *in vitro* work investigated the potential for PRGF and Manuka honey/MH to stimulate the activity of hDF, hPMECs, and macrophages in ways that would provide insight into their specific roles in the healing process. Cell proliferation, chemotaxis, cytokine release, and ECM production were all tested in the presence of PRGF and Manuka honey media supplements in order to determine what response, if any, each cell type had to the additives and which additive/concentration proved to be most efficacious.

PRP, in its liquid form has been used previously as a media supplement in the culture of a number of cell lines (hDFs, bone marrow and adipose derived mesenchymal stem cells, patellar tendon fibroblasts, anterior cruciate ligament fibroblasts) [34, 48–56]. These studies have all documented that the addition of PRP to culture media resulted in increased rates of cell proliferation or was at minimum effective in maintaining rates of cell proliferation in the place of a traditional serum supplement. In this study, rather than utilize PRP, a dry PRGF powder was created by subjecting PRP to a freeze-thaw-freeze process and subsequent lyophilization. The creation of this powder allows for easier handling of the material, while further concentrating its contents through the removal of the liquid plasma portion. Despite this processing, it has been previously demonstrated that PRGF retains its bioactivity and can successfully impact cellular activity [41, 42]. The use of the PRGF in this study clearly demonstrates effectiveness in stimulating cell proliferation, chemotaxis, matrix production, and angiogenesis. The presence of PRGF-supplemented media promoted significant increases in the proliferation of hDFs and hPMECs at lower concentrations, while higher concentrations resulted in the formation of a clot that impeded accurate cell quantification. While this clotting action was definitely a hindrance to obtaining accurate cell proliferation/chemotaxis results in this study, it also demonstrated the retained inherent activity of the PRGF powder. While determining the clotting potential of the PRGF was not the intent of this study, this result was seen as an added benefit and may prove beneficial as a hemostatic product. Overall, the results of the use of PRGF are similar to what has been previously published with PRP. Namely, PRP can significantly increase the activity of a number of different cell types. The statistically significant increases in hDF proliferation and migration, as well as the increases in hPMEC proliferation and chemotaxis clearly demonstrate that the cytokines present in the PRGF retain their functionality after processing.

As noted, Manuka honey has been re-gaining popularity among the wound healing community [3, 5, 9–11, 57]. The rather unique properties possessed by the active *Leptospermum* honeys are all highly beneficial in tissue regeneration. To evaluate the hypothesis that the low pH of the Manuka honey would effectively activate PRGF-derived growth factors, the impact of Manuka honey supplemented media on three distinct cell lines was evaluated. The modified Western Blot performed demonstrated the presence of activated TGF-*β* derived from the combination of MH and PRP. While this test did not provide a quantitative assessment of how much active TGF-*β* was present, it offered a side by side comparison of the two treatment methods and showed them to be nearly identical. The presence of this activated TGF-*β*, and potentially other acid activated cytokines, may have played a major role in the impact that PRGF+MH had in accelerating cellular activity.

Interestingly, throughout the study, there were no significant differences between the two honey products tested: the pure Manuka honey and the medical grade MH. This would indicate that the filtration and sterilization process used to make the MH has no ill effects on its efficacy. The only significant difference experienced between the two products was seen in the macrophage inflammation response, where TNF-*α* release was significantly increased in the 1% Manuka honey on days 1 and 4. This makes sense as the release of TNF-*α* commonly indicates an inflammatory response and an M1 macrophage phenotype [29–32, 58, 59]. It is completely possible, and highly likely, that the pure Manuka honey may contain some amount of allergens or bee byproducts that could induce an inflammatory response. It would then make sense that the sterilized MH product would be allergen and bee byproduct free. As small amounts of IL-10 were produced by the macrophages during this study, that may indicate that the macrophages expressed an anti-inflammatory phenotype (regulatory macrophage). Looking at macrophage phenotype as a highly plastic, continuous spectrum, the regulatory macrophage serves as a transitional bridge through the wound healing cascade between the M1 and the wound healing macrophages (M2). The presence of quantifiable IL-10, and a lack of quantifiable VEGF, would indicate that the macrophages resided within this regulatory phenotype and had not progressed to the M2 phenotype [31, 32]. It may be possible that longer duration culture would have initiated the expression of the M2 phenotype and possible VEGF production, and this may be investigated in the future.

Another interesting result provided by these *in vitro* cell-honey interaction studies was the potential for the Manuka honey products to become cytotoxic. In nearly all the studies performed, with all three cell types utilized, concentrations of Manuka honey or MH of 5% v/v and above resulted in the death of the cells to be studied. It is hypothesized that this cytotoxicity was due to the acidic pH and the closed nature of the *in vitro* studies performed. To the authors' knowledge, there have been no reports of any Manuka products being cytotoxic when used clinically. In a clinical setting, there are far better mechanisms for diffusion, nutrient exchange, and waste removal than there are in an *in vitro* study, where cells are essentially sequestered and highly responsive to changes in local pH. For this reason, it is highly likely that this preliminary *in vitro* study may not demonstrate the true potential of using a Manuka honey product in a wound healing capacity and further *in vivo* and *in situ* studies will be required. It may be possible that the promising results achieved here with low concentrations of honey may be surpassed with higher honey concentrations in an *in vivo* setting, where the effects of localized pH changes will be less inhibitory.

While the presence of PRGF and Manuka/MH supplemented media proved effective in stimulating a positive cellular response individually, the use of PRGF+MH media demonstrated a further increase in cellular activity. This impact was seen most clearly in the hDF-based studies, and the *in vitro* wound healing assay study, where the PRGF+MH media supplements resulted in statistically significant increases in cell proliferation and migration. As previously stated, this increase in cellular response may be due to the presence of physiologically activated growth factors such as TGF-*β*. However, this may only be a small part of the overall potential for the PRGF+MH combination to increase cellular activity. It is entirely possible that since the individual PRGF and MH components each provide necessary pieces of the tissue regeneration puzzle, the combination of PRGF+MH may provide an ideal combination to stimulate wound healing. However, determining the exact mechanism of cell stimulation is beyond the scope of this preliminary study and will need to be evaluated in future work. While the PRGF+MH yielded results that exceeded the authors' expectations in the hDF-specific studies, the angiogenesis studies proved to be less conclusive. This would signify that the impact on the initiation of angiogenesis was due to the presence of PRGF alone and was not influenced by the addition of the Manuka/MH. This may indicate that the rate of sprout formation and angiogenesis are highly dependent upon the presence of available VEGF, readily found in PRGF, in which case the presence of honey would not offer much benefit. However, it was anticipated that the pH of the honey products alone may be enough to induce sprout formation, as it has been documented that an acidic environment may promote angiogenesis [2, 4]. Therefore, as previously mentioned, these results may be indicative of the need for an *in vivo* angiogenesis model.

In summary, PRP/PRGF capitalizes on the body's own initial healing component, the platelet, and concentrating them to thereby concentrate their inherent growth factors and “jump start” the healing process. In the lyophilized powdered form, the PRGF powder becomes more versatile (i.e., able to be stored in a vacuum sealed package) and less messy than the liquid PRP while retaining its potent bioactive properties. Additionally, it may be possible that this powdered PRGF, further enhanced through the addition of a MH product, may be carried by first responders or military personnel to be used at the time of injury. It is anticipated that not only will the application of a PRGF and Manuka honey product accelerate the healing process, as demonstrated with the *in vitro* results described here, but may also promote coagulation and provide antibacterial properties to a wound in the field. It may also be possible that such a combination product could prove to be highly effective in treating chronic wounds, where cellular senescence, low tissue friability, and biofilm formation are all concerns. Having an essentially “all natural” product known to be highly antibacterial, yet capable of stimulating a number of different cell types, at a patient's bedside capable of advancing the wound from a state of chronic inflammation to a more conducive healing state would be of great benefit to the treatment of chronic wounds.

## 5. Conclusion

In this study, it was demonstrated that a number of cell types critical to dermal regeneration (hDF, hPMEC, and macrophages) are capable of being positively influenced by the presence of PRGF and Manuka honey media supplements. Based upon these results, it is apparent that PRGF has the capacity to enhance cellular chemotaxis, mitogenesis, ECM production, and angiogenesis. This bioactivity can be further augmented by combining PRGF with MH, potentially due to the increased presence of growth factors that have become physiologically activated. The precise nature of this enhancement is not currently understood and may stem from a number of attributes unique to the two materials acting concomitantly: the presence of necessary sugars, growth factors physiologically activated through Manuka honey pH, direct pH effects on cells, the presence of Vitamin C, and others. While further research is needed to definitively determine the method of action in which the PRGF and MH permutation promote cellular activity, the use of this combination of materials has great potential from a regenerative medicine perspective; being able to apply a lyophilized PRGF powder, with or without a Manuka honey product, to the site of a dermal wound at the time of injury may not only assist with clotting but may also accelerate closure and healing of the wound. Ongoing work includes *in vivo* evaluations of wound healing and angiogenesis in small animal models, *in vitro* antibacterial investigation, as well as *in vitro* work with a human keratinocyte cell line.
